# *Carica papaya* Leaf Extract Silver Synthesized Nanoparticles Inhibit Dengue Type 2 Viral Replication In Vitro

**DOI:** 10.3390/ph14080718

**Published:** 2021-07-26

**Authors:** Antonia Windkouni Bere, Omuyundo Mulati, James Kimotho, Florence Ng’ong’a

**Affiliations:** 1 Department of Molecular Biology and Biotechnology, Pan African University Institute for Basic Sciences, Technology and Innovation, Nairobi P.O. Box 62000-00200, Kenya; 2 Department of Biomedical Sciences and Technology, Technical University of Kenya, Nairobi P.O. Box 52428-00200, Kenya; omuyundo.mulati@gmail.com; 3 Innovation and Technology Transfer Division, Kenya Medical Research Institute, Nairobi P.O. Box 54840-00200, Kenya; james@drugindex.co.ke; 4 Department of Biochemistry, Jomo Kenyatta University of Agriculture and Technology, Nairobi P.O. Box 62000-00200, Kenya; fngonga@jkuat.ac.ke

**Keywords:** dengue virus, *Carica papaya*, silver nanoparticles, antiviral activity

## Abstract

The current global occurrence of dengue infection annually is approximately 400 million, with a case fatality rate of 2.5%. However, there are no antiviral agents. *Carica papaya* leaf extract is known for its medicinal value, due to the presence of organic compounds that possess antimicrobial, anti-inflammatory, and antioxidant activities. This study determined the anti-dengue effect of *C. papaya* leaf extract silver synthesized nanoparticles. In this study, aqueous and non-aqueous extractions were carried out, followed by the synthesis of silver nanoparticles as well as characterization through Fourier transform infrared spectroscopy (FTIR) and scanning electron microscopy. The in vitro anti-dengue effect was evaluated using a focus reduction neutralization test on kidney Vero E2 cell lines. In silico studies involved molecular docking to determine the potential interactions between the bioactive compounds in *C. papaya* leaf extract and the viral NS5 protein. *C. papaya* leaf methanol extract silver synthesized nanoparticle was the most promising with an IC_50_ of 9.20 µg/mL. Molecular docking showed 5,7 dimethoxycoumarin as the best ligand, with binding energy of −7.75 kcal/mol, indicating high affinity for the NS5 protein. These results highlight that *C. papaya* leaf methanol extract silver synthesized nanoparticles could be used to inhibit dengue virus type 2 viral replication. However, we recommend further studies to determine their toxicity and the safety profiles.

## 1. Introduction

Dengue is a mosquito-borne viral disease caused by dengue virus (DENV), a member of the Flaviviridae family. There are four serotypes of dengue virus: DENV-1, DENV-2, DENV-3, and DENV-4; however, DENV-2 appears to cause more epidemics [1]. The virus infects approximately 400 million people annually around the world, especially in the tropical and sub-tropical regions [2,3]. With a case fatality rate of 2.5% and 1300 disability-adjusted life years (DALYs) per million, dengue infections cause a significant economic burden to public health systems in the endemic countries. Currently, with no effective antiviral agents, treatment is essentially supportive and symptomatic [4]. Development of therapeutic strategies is still an urgent need to prevent dengue fatalities [5]. The use of herbal medicine in disease management has long been in existence and played a vital role in the treatment of various infections, including dengue [6,7,8]. Several studies have shown that herbal extracts could be useful in the treatment of dengue. For instance, extracts of *Scutellaria baicalensis* (a traditional Chinese medicinal herb), bioflavonoids, and green synthesized nanoparticles using the *Moringa oleifera* seed extract have been shown to inhibit dengue viral replication in vitro [9,10,11]. 

*C. papaya* is an herbaceous succulent plant, belonging to the Caricaceae family. Different parts of the plant (fruit, seed, and leaves) are employed in the treatment of different human and veterinary diseases in various parts of the world. *C. papaya* leaf extract is known for its medicinal capabilities, due to the presence of a number of important organic compounds that possess antimicrobial, anti-inflammatory, and antioxidant activities [11,12]. Studies have reported that the leaf extracts of *C. papaya* contain bioactive compounds like the *papain* enzyme, alkaloids, flavonoids, saponins, and tannins [13]. The leaves are commonly used in the treatment of varied forms and stages of medical complications (arthritis, digestive disorders hypertension, malaria, and ringworms) [14] and are of particular importance is in the treatment of dengue virus infection [13,15]. *C. papaya* leaf extracts help to increase platelet levels and have demonstrated definitive beneficial effects in patients with dengue infection [16,17]. Green synthesized silver nanoparticle drug delivery systems hold a high level of promise in the ever-evolving drug design and delivery systems [10].

Virtual screening has enabled the prediction of drug activity against the specific targets and shortened time spans for drug discovery [18]. Virtual screening uses molecular docking to predict the best binding orientations for ligands. These docking data provide the correct conformation of a ligand–receptor complex and its binding affinity, in terms of binding free energy [19]. Due to the diversity of phytochemicals present in plants, this study employed molecular docking of key *C. papaya* phytochemical constituents, such as chlorogenic acid, dimethoxy-coumarin, kaempferol, protocatechuic acid, and quercetin to determine their interaction with the NS5 protein of dengue virus type 2, the most conserved non-structural proteins known to replicate the viral RNA genome [20]. The study also investigated the in vitro activity of *C. papaya leaf* extracts silver synthesized nanoparticles against DENV-2.

## 2. Results

### 2.1. UV-Vis Spectrophotometric Analysis

**Figure 1:**
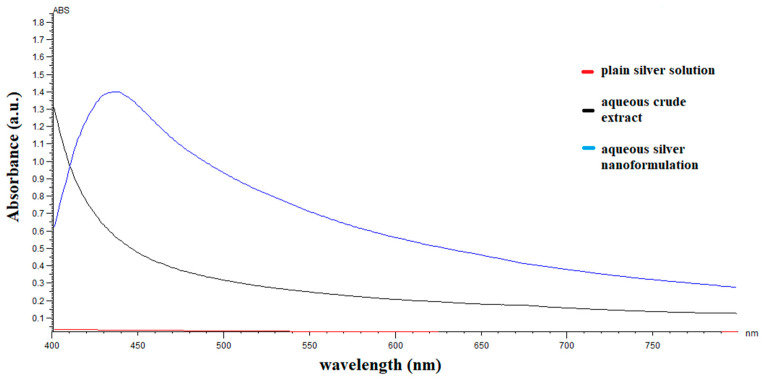
UV–visible spectra of aqueous extract of *C. papaya* leaf-silver nanoparticles and absorbance peak noted at 450 nm.

**Figure 2:**
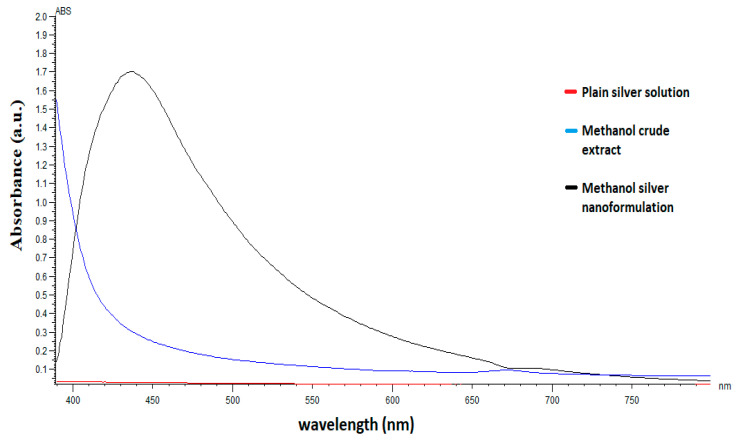
UV–visible spectra of methanol 70% extract of *C. papaya* leaf-silver nanoparticles and absorbance peak noted at 450 nm.

The UV absorption spectrometric analysis of the *C. papaya* leaf extract synthesized silver nanoparticles showed the absorbance spectra at 450 nm for the aqueous and methanolic extracts, suggesting the bioreduction of silver nitrate into silver nanoparticles (Figure 1 and Figure 2, respectively).

### 2.2. FTIR Analysis

**Figure 3:**
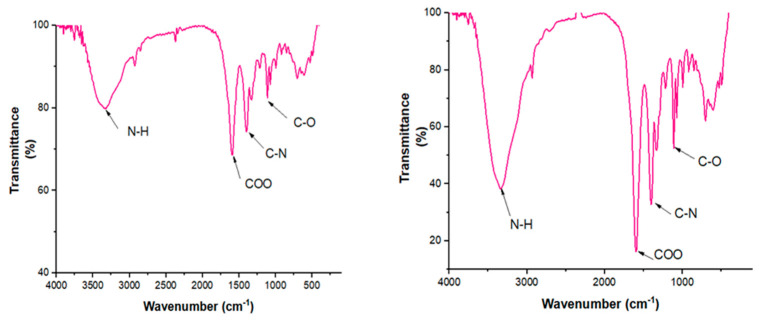
FTIR spectra of aqueous and 70% methanol extract of *C. papaya* leaf silver synthesized nanoparticles.

The FTIR analysis spectrum showed sharp absorbance between 4000 and 500 cm^−1^. The picks in the spectrum showed the presence of proteins and metabolites possessing functional groups such as alcohols, ketones, aldehydes, or carboxylic groups in the synthesized silver nanoparticles (Figure 3).

The absorption peaks at 3571, 1326, and 3317 cm^−1^ were observed in the aqueous leaf extract due to OH stretching vibration. Absorbance at 1596 cm^−1^ is accredited to an alkyne group indicated by the presence of an aromatic ring. The peaks at 1218, 1110, and 1072 cm^−1^ correspond to the C–O stretching of alcohol and hydroxy compounds (phenols). For the methanol extract, the absorption peaks at 3340, 1404, and 702 cm^−1^ were observed in the leaf extract due to OH stretching vibration. Absorbance peaks at 2931, 1334, 995, 918, and 848 cm^−1^ were characteristics to an alkyne group indicating the presence of an aromatic ring. The picks at 1211, 1110, and 1072 cm^−1^ corresponded to the C–O stretching of alcohol and hydroxy compounds (phenols).

### 2.3. Scanning Electron Microscopy

**Figure 4:**
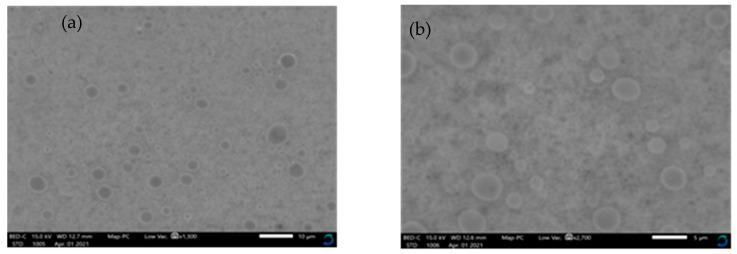
Scanning electron microscopy micrographs of synthesized *C. papaya* leaf extracts silver nanoparticles (spherical) on (**a**) 10 µm scale and on (**b**) 5 µm scale.

The scanning electron microscopy analysis showed particle sizes between 10 and 35 nm for both the aqueous extract and methanolic extract, with a spherical morphology (Figure 4).

### 2.4. Total Flavonoid and Phenolic Content Determination

The total flavonoid and phenolic content of the *C. Papaya* leaf methanolic extract silver synthesized nanoparticles were 179.70 ± 9.42 and 92.02 ± 0.53 mg of rutin/mg, respectively, indicating the presence of flavonoids and phenolic compounds in *C. papaya* leaf extracts (Table 1).

### 2.5. Docking Interactions of Selected Bioactive Compounds with Dengue Type 2 Virus Non-Structural Protein 5

**Figure 5:**
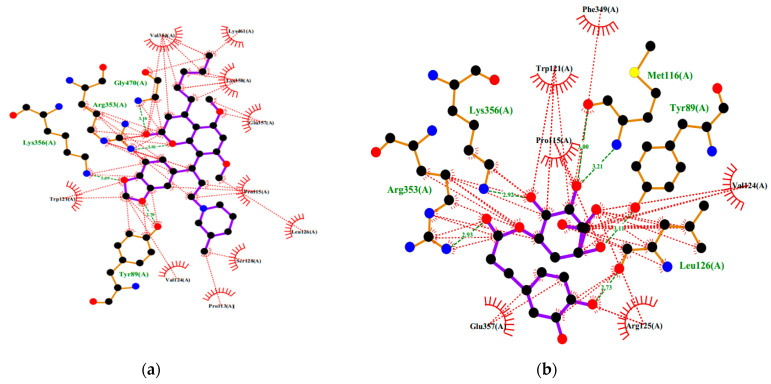

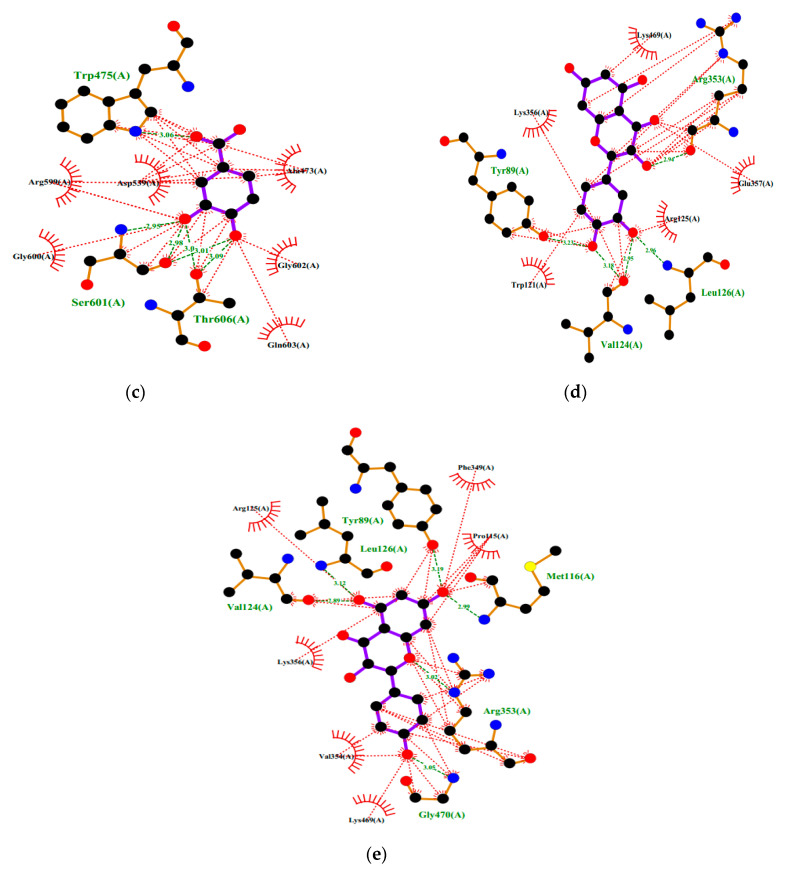
Docking complexes of the bioactive compounds from *C. papaya* leaf extracts against DENV-2 NS5 protein; (**a**) 5,7- Dimethoxycoumarin, (**b**) E-Chlorogenic acid, (**c**) Protocatechuic acid, (**d**) Quercetin, and (**e**) Kaempferol.

In silico studies focused on selected bioactive compounds of *C. papaya* extracts (5,7 dimethoxycoumarin, E-chlorogenic acid, Quercitin, Kaempferol, and Protocatechuic acid) against DENV-2 NS5 protein, all the five bioactive compounds exhibited acceptable binding affinity between −6.34 and −7.75 kcal/mol, with the best ligand being 5,7 dimethoxycoumarin (Table 2). The docking complexes and interactions of the bioactive compounds against DENV-2 NS5 protein revealed interactions with amino acid residues in different domains of the protein (Table 2 and Figure 5).

### 2.6. In Vitro Assay

**Figure 6:**
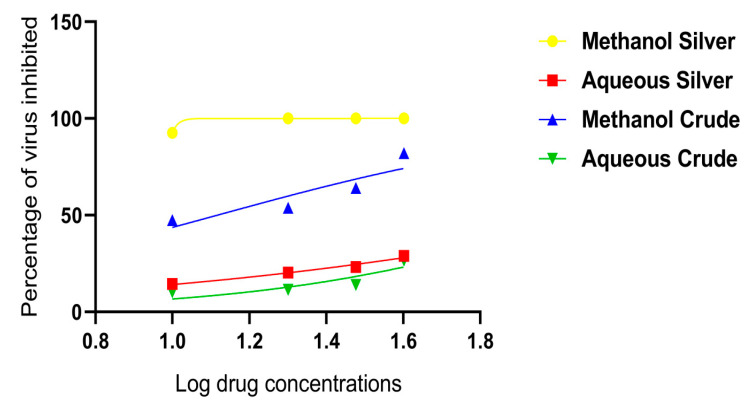
Percentage inhibition of the different *C. papaya* leaf extracts against cultured dengue virus type 2.

To determine the IC_50_ value of each sample, a regression curve of the extract concentration-DENV-2 inhibition percentage was made using graph pad prism 8.4.3. The antiviral activity of the synthesized silver nanoparticles and crude extract of *C. papaya* leaves tested against DENV-2 indicated that the *C. papaya* methanol leaf extract silver synthesized nanoparticles were the most promising, with an IC_50_ of 9.20 µg/mL (Table 3 and Figure 6).

**Figure 7:**
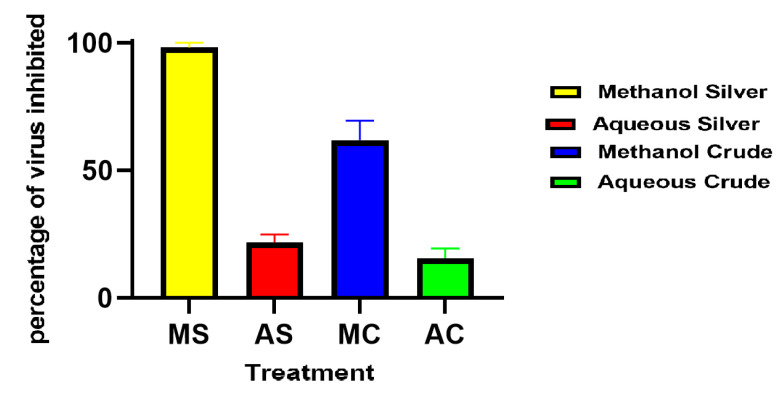
Percentage inhibition of dengue virus type 2 with the different extracts of *C. papaya.* Values represent mean ± SE (P0.05, *n* = 4).

Statistical analysis at a *p* < 0.05 showed that the percentage of inhibition of the extracts silver synthesized nanoparticles against dengue virus type 2 was significantly different from the crude extracts viral percentage inhibition. A similar observation was also made for the aqueous and the methanolic extracts (Figure 7).

## 3. Discussion

The synthesis of silver nanoparticles using plant extracts is getting more attention, due to their application in biomedical sciences such as anti-parasitic, anti-malarial, bactericidal, fungicidal, and anti-viral activity [21]. In this study, the AgNPs were synthesized using aqueous and methanol extracts of *C. papaya*. Initially, the formation of AgNPs was confirmed by observing the color change of the reaction mixture. Within 15 min of the exposure of the *C. papaya* extract, the color of silver nitrate (AgNO_3_) solution changed from transparent to a dark brown color which is a primary indication of AgNPs production. This color change was observed due to the reduction of Ag^+^ ions in the solution of AgNO_3_ into AgNPs. The color change was in line with previous studies that demonstrated the appearance of a brown color, due to excitation of surface plasmon vibrations with the synthesized AgNPs [22]. Furthermore, the production of AgNPs was confirmed by UV-Visible spectroscopic analysis, which quantifies the absorption spectra. The UV-visible spectrometry showed a sharp peak at 450 nm, confirming the formation of silver nanoparticles. This is in range with previously reported studies on the synthesis of AgNPs from *C. papaya* [19,23,24].

From the FT-IR analysis, the presence of hydroxyl, aromatic ring, alcohol, and amide groups in the *C. papaya* leaves extracts is confirmed by the picks observed. The FT-IR analysis suggested that proteins were involved in the capping and stabilization of the synthesized silver nanoparticles [25]. The C-O picks observed in the methanolic extract suggested that more phenols compounds were found in the methanolic nanoparticles, as compared to aqueous extract.

Through scanning electron microscopy, the synthesized nanoparticles showed particle sizes ranging from 10 and 35 nm, which may confer the ability to penetrate the cells. This is in agreement with previous studies by Banala et al., who reported average nanoparticle sizes between 5 and 40 nm, with a spherical morphology, and Sar & Upadhayay, who also reported spherical shaped nanoparticles with varied sizes ranging from 5 to 50 nm [26,27].

The quantification of flavonoids and phenols content in the extracts confirmed the presence of flavonoids and phenolic compounds in the *C. papaya* leaves and indicated that the total flavonoid and phenolic content of the synthesized nanoparticles was higher, compared to the crude extracts. Similar to our results, Abdel-Aziz and Salari reported a higher total phenol and flavonoid content in the synthesized AgNPs of *Prosopis farcta* fruit extract and *Chenopodium murale* leaf extract, respectively [28,29]. In addition, the content of the flavonoids and phenolic compounds in the aqueous extract of *C. papaya* leaves was lower, compared to the methanolic extract. This is due to the fact that the cell wall has non-polar character and, therefore, degraded efficiently in organic solvent compared to water, enhancing the extraction process [21].

The current study shows that both the methanolic crude and methanolic silver synthesized nanoparticles from *C. papaya* leaf extracts had high antiviral activities against dengue virus type 2 with IC_50_ values of 13.09 µg/mL and 09.20 µg/mL, respectively. This may be due to the phytochemical content of the different extracts. Studies reported that flavonoids are a major group of phenolic compounds with antiviral properties [30]. Parthiban et al. reported that the crude aqueous extracts of *C. papaya* leaves possess alkaloids, saponins, tannins, and terpenoids [31]. In addition to the phytochemicals present in *C. papaya* aqueous extracts, organic extracts also possess other reducing sugars [32]. This observation indicates that the difference in activity could be due to the differences in the phytochemical composition of the extracts. According to our results, the biosynthesized silver nanoparticles of *C. papaya* extract had enhanced the antiviral activity against dengue type 2 virus. This is in line with previous studies that had proven nanomaterials to be more efficient for drug delivery [10,33,34].

The gas chromatography–mass spectrometry analysis of previous studies states that the leaves of *C. papaya* possess phenolic compounds such as quercetin, protocatechuic acid, 5,7 dimethoxycoumarin, chlorogenic acid, and kaempferol [35] used in our in silico study. This study revealed possible interactions between the various bioactive compounds present in the *C. papaya* leaf extract and both the N- and C-terminal domains of the viral NS-5 protein. The NS-5 protein N-terminal domain possesses the Cap-0 specific (nucleoside-2’-O-)-methyltransferase, which catalyzes the methylation of Cap-0 at the 2’-hydroxyl of the ribose of the first nucleotide, using S-adenosyl-L-methionine as the methyl donor. This reaction is the last step in mRNA capping, the creation of the stabilizing five-prime cap on mRNA. The C-terminal domain is the Flavivirus RNA-directed RNA polymerase, which produces a polyprotein from the ssRNA genome. Thus, the viral inhibition observed via the focus reduction neutralization test could be due to the inhibition of viral replication.

## 4. Materials and Methods

### 4.1. Chemicals and Reagents

Silver nitrate (Sigma-Aldrich, Burlington, VT, USA) and plant extract (*C. papaya* leaves) were used. Other chemicals and reagents used in this study were of laboratory grade. Vero cells (African green monkey kidney epithelial cells) and Dengue virus type 2 strains were kindly provided by the Kenya Medical Research Institute (KEMRI).

### 4.2. Collection of Plant Materials

Fresh green leaves of *C. papaya* were collected from Gatundu South Constituency (−0.97566, 36.84921) in Kenya. The collected leaves were transported to the laboratory in sterile bags. Identification and characterization of the collected materials was performed at Jomo Kenyatta University Agriculture and Technology by a well-trained botanist. A sample voucher specimen No. AWB-JKUATBH/001C-2020 was deposited at JKUAT herbarium.

### 4.3. Sample Preparation and Extraction

The leaves with black or yellow spots and those appearing old were discarded. The selected *C. papaya* leaves were washed twice with distilled water to remove sand and other particles or contaminants. After washing, the leaves were cut into small pieces and spread for drying under shade. The dried leaves were ground using a blender. In the laboratory, both aqueous (distilled water) and organic (methanol 70%) extraction techniques were used.

For aqueous extraction, 10 g of powdered *C. papaya* leaves was mixed with 100 mL of hot sterile distilled water and allowed to stand for 30 min. The extract was then filtered using Whatman no.1 filter paper, the filtrate was freeze-dried (MRC freeze dryer; FDL-10N-50-BA), and the powder stored at 4 °C until use.

For the organic solvent extraction, 10 g of the powdered *C. papaya* leaves was macerated in 50 mL of 70% methanol for 72 h, followed by the filtration of the solution using Whatman no.1 filter paper. The filtrate was concentrated using a rotary evaporator (Labtech DAIHAN, VP30, EV11, Sorisole, Italy) (rpm 40, temperature 60 °C) to remove all the methanol in the extract. The extract with only water remaining was freeze-dried and the powder stored at 4 °C for further use.

Silver nitrate (AgNO_3_) was used as the precursor for the synthesis of silver nanoparticles. One millimole (1 mM) of the aqueous solution of silver nitrate was prepared using distilled water. One (1) g of the powdered leaf extracts, from aqueous and methanol 70% extraction, were weighed and diluted using 50 mL of distilled water. This was then added to the AgNO_3_ solution in the ratio of 1:4, as described by Banala et al. [26], with slight modification for the bio-reduction process and protected from light using aluminum paper at 30 °C. The reaction was performed for 24 h with constant stirring. The bioreduction of the Ag^+^ was accompanied by a change in color from greenish to dark brown. The silver nanoformulated solutions were freeze-dried in a vacuum chamber (MRC, MRC freeze dryer, FDL-10N-50-BA). The lyophilized powder was stored at 4 °C for characterization and in vitro assays.

### 4.4. Characterization of C. papaya Leaf Extract Silver Synthesized Nanoparticles

#### 4.4.1. UV–Visible Spectrometric Analysis

The reduction of silver ions (Ag^+^) by *C. papaya* leaf extracts was monitored by periodic scanning (10 min to 60 min) using the UV spectrophotometer (UV—vis spectrophotometer 6800, Jenway) at a wavelength between 200 nm and 800 nm.

#### 4.4.2. Fourier Transform Infrared (FTIR) Analysis

The Fourier transform infrared analysis was utilized to evaluate the key functional groups present. Two milligrams of the sample were mixed with 100 mg KBr (FT-IR grade) and then compressed to prepare a salt disc (3 mm diameter). The disc was immediately kept in the sample holder and the FT-IR spectra were recorded in the absorption range between 4000 and 500 cm^−1^. All investigations were carried out using a Shimadzu FT-IR spectrometer (FTIR Shimadzu 8400, Kyoto, Japan).

#### 4.4.3. Scanning Electron Microscopy (SEM)

The particle size and surface morphology of the nanoparticles were confirmed using a Scanning Electron Microscope (JCM-7000 Neoscope benchtop SEM, Jeol, Tokyo, Japan), performed at Fortification lab, JKUAT. Thin films of the sample were prepared on a carbon-coated, copper grid by placing a very small amount of the sample on the grid; extra powder was removed using a blotting paper and then the films on the SEM grid were analyzed.

### 4.5. Determination of Total Phenolic Content (TPC)

Total phenolic content in the *C. papaya* leaf extracts was measured spectrophotometrically via the Folin–Ciocalteu colorimetric method, described by Makkar [36], using gallic acid as the standard and expressing results as the gallic acid equivalent (GAE) per gram of sample. Different concentrations (2.5–12.5 µg/mL) of gallic acid were prepared in methanol. Aliquots of 50 µL of the test sample and each sample of the standard solution were taken, mixed with 0.5 mL of 1N Folin–Ciocalteu reagent and 2.5 mL of a saturated solution of 5% sodium carbonate. The tubes were incubated for 40 min in the dark at room temperature. The absorbance was taken at 725 nm using methanol as blank. All the samples were analyzed in triplicate.

### 4.6. Determination of Total Flavonoid Content

The total flavonoid content of the extract was determined by aluminum chloride colorimetric assay, described by Zhishen [37]. Briefly, 0.5 mL aliquots of the extract and standard solution (16–80 µg/mL) of rutin were added with 0.5 mL of distilled water and, subsequently, with 0.15 mL of sodium nitrite (5% NaNO_2_, *w*/*v*) solution and mixed. After 6 min, 0.15 mL of (10% AlCl_3_, *w*/*v*) solution was added. The solutions were allowed to stand for a further 6 min and after that, 2 mL of sodium hydroxide (4% NaOH, *w*/*v*) solution was added to the mixture. The final volume was adjusted to 5 mL, with an immediate addition of distilled water, mixed thoroughly and allowed to stand for another 15 min. The absorbance of each mixture was determined at 510 nm against the same mixture, but without the leaf extract as a blank. Total flavonoid content was determined as the mg rutin equivalent per gram of sample, with the help of the calibration curve of rutin. All determinations were performed in triplicate.

### 4.7. Cell Culture and Virus Propagation

The kidney epithelial Vero cells were grown at 37 °C with 5% CO_2_ in minimum essential medium (MEM) supplement, 10% fetal bovine serum, 2% l-Glutamine, 2% Streptomycin/amphotericin B, 7.5% sodium biocarbonate, and 1% non-essentiel amino acid, as described by Zandi [38].

Dengue virus type 2 was propagated in the Vero cell line at 37 °C in 5% CO_2_. The stock of DENV-2 was obtained by adding 200 µL of DENV-2 to confluent Vero cells in a T25 cm^2^ flask and gently shaken for 1 h to maximize the viral adsorption to the cells. After which, 5 mL of fresh growth medium was added prior to incubation at 37 °C with 5% CO_2_ for 9 days. Culture supernatant was harvested and centrifuged at 3000× g for 15 min. The supernatant was collected and stored at −80 °C.

### 4.8. Determination of Antiviral Activities

A focus assay was used to determine the viral titers before performing the focus reduction neutralization test. Ninety-six-well plates were seeded at 2.5 × 10^4^ Vero cells per well and placed in a 37 °C incubator in an atmosphere of 5% CO_2_ for 24 h. Ten-fold serial dilutions of the virus were inoculated in triplicate on the 96-well plates and incubated at 37 °C, in an atmosphere of 5% CO_2_ for 90 min, for virus adsorption. After adsorption the wells were overlaid with 1.25% methylcellulose in maintenance media and incubated at 37 °C with 5% CO_2_ for three days. Infected cells were immunostained. For immunostaining, the methylcellulose overlay medium was removed and the cell monolayers were fixed with 5% formaldehyde solution for three hours at room temperature. After three h, the formaldehyde was removed and the wells were then washed one time, gently, with 1× phosphate buffered saline (PBS ^(−)^). One (1)% Nonidet^R^ P 40 Substitute (NP-40) was added and incubated at room temperature for 20 min then washed three times with PBS ^(−)^, followed by incubation with blocking buffer at room temperature for 30 min. The cells were washed three times with PBS ^(−)^ and incubated with 1000× diluted high dengue type 2 IgG titer patient pooled serum at 37 °C for 1 h. After washing three times with PBS ^(−)^, incubation was done with 500× diluted HRPO conjugated anti-human IgG goat serum (AQL:A110PD) at 37 °C for one hour. DAB substrate was added after the wells were washed three times with PBS ^(−)^ and incubated for 20 min. Immunostained plates were rinsed with double-distilled water and allowed to air dry before counting the foci under a stereo-microscope.

### 4.9. Dengue Focus Reduction Neutralization Test

For the antiviral assays, a stock solution was prepared by dissolving 0.2 g of the extract in 20 mL of dimethyl sulfoxide (DMSO). The stock solution was filtered, sterilized (0.20 µm pore), and further diluted with a culture medium to the desired concentration for the assays.

The in vitro antiviral assay was initiated by seeding 2.5 × 10^4^ cells/well into each well of 96-well plate and left to incubate at 37 °C with 5% CO_2_ for 24 h. The dengue virus was diluted with maintenance media at 10^−2^. The stock solution of the different samples of *C. papaya* was diluted to obtain the following concentrations of 80 µg/mL, 60 µg/mL, 40 µg/mL, and 20 µg/mL. An equal volume of dengue virus type 2 suspension of approximately 120 focus-forming units/mL was added to each diluted extract and the virus-extract mixture was incubated at 37 °C in an atmosphere of 5% CO_2_ for 60 min to enable neutralization to occur. The final concentrations of the extracts tested against dengue virus were 40 µg/mL, 30 µg/mL, 20 µg/mL, and 10 µg/mL. The cell culture medium was then aspirated from the 96-well plates with pre-formed Vero cell monolayers and the virus-extract mixture (100 µL/well) was inoculated into each well and incubated at 37 °C in an atmosphere of 5% CO_2_ for 90 min to enable the non-neutralized dengue virus to adsorb onto Vero cells. After adsorption, the wells were overlaid with 1.25% methylcellulose in maintenance media and incubated at 37 °C with 5% CO_2_ for three days. Infected cells were immunostained and the assays were conducted alongside controls.

### 4.10. Molecular Docking

To determine the possible interaction with the viral NS5 protein, the structure of *C. papaya* bioactive compounds (Kaempferol, 5,7-Dimethoxycoumarin, quercetin, E-chlorogenic acid, and protocatechuic acid) were retrieved from the PubChem database in sdf format. The NS5 crystal structure was also retrieved from the protein data bank using the 5zqk accession number. Molecular Graphics Laboratory (MGL) Tools (version 1.5.4) were used to prepare the protein; uscf-chimera was to prepare the chemicals and to analyze the docking output. The molecular docking was performed using the Swissdock web server (http://www.swissdock.ch/docking, 22 November 2020). The grid box was set at X = 36.49, Y = 4.79, and Z = 42.28. The grid box layout was set at 60 × 60 × 60.

## 5. Conclusions

Our results showed that the *C. papaya* leaves’ methanol extract silver synthesized nanoparticles highly inhibited DENV-2 replication in vitro with a viral inhibition percentage greater than 90%, at lower IC_50_ values of 9.20 µg/mL. The results also highlight the possibility of the synergistic activities of the different bioactive compounds present in *C. papaya* leaf extract, through interactions with both conserved domains of the viral NS5 protein. We recommend further studies to determine their toxicity and the safety profiles in vivo.

## Figures and Tables

**Table 1:** Total phenolic and flavonoid contents of synthesized *C. papaya* leaf extracts silver nanoparticles.

Samples	Flavonoids (mg rutin/g Extract)	Total Phenolics (mg GAE/g Extract)
Aqueous extract AgNPs	119.84 ± 13.16	5.86 ± 0.16
Methanol extract AgNPs	179.70 ± 9.42	92.02 ± 0.53
Crude aqueous extract	43.73 ± 2.36	2.76 ± 0.13
Crude Methanol extract	56.39 ± 4.09	53.24 ± 8.18

**Table 2:** Binding interactions of DENV-2 NS5 with specific bioactive compounds of *C. papaya* leaf extracts.

Compounds	Residues	NS5 Domain	Hydrogen Bonding	Bond Length (Å)	Binding Energy (kcal/mol)
Protocatechuic acid	Trp 475	C-ter	1	3.06	−6.65
	Ser 601	C-ter	3	2.95, 2.98, 3.01	
	Thr 606	C-ter	2	3.0, 3.09	
E-Chlorogenic	Lys 356	C-ter	1	2.92	−7.35
	Arg 353	C-ter	1	2.93	
	Met 116	N-ter	2	3.00, 3.21	
	Tyr 89	N-ter	1	3.11	
	Leu 126	N-ter	1	2.73	
Quercetin	Arg 353	C-ter	1	2.94	−7.07
	Tyr 89	N-ter	1	3.23	
	Val 124	N-ter	2	3.18, 2.95	
	Leu 126	N-ter	1	2.96	
Kaempferol	Tyr 89	N-ter	1	3.19	−7.01
	Met 116	N-ter	1	2.99	
	Leu 126	N-ter	1	3.12	
	Val 124	N-ter	1	2.89	
	Arg 353	C-ter	1	3.02	
	Gly 470	C-ter	1	3.05	
5,7 Dimethoxycoumarin	Gly 470	C-ter	1	3.1	−7.75
	Arg 353	C-ter	1	3.01	
	Lys 356	C-ter	1	3.09	
	Tyr 89	N-ter	1	2.7	

**Table 3:** Percentage viral inhibition of the different *C. papaya* leaf extracts.

Extracts	Conc. 10 µg/mL	20 µg/mL	30 µg/mL	40 µg/mL	IC50 (µg/mL)
Methanol AgNPs	92.59% ± 0.18	100.00% ± 0.0	100.00% ± 0.0	100.00% ± 0.0	9.20
Aqueous AgNPs	14.49% ± 0.24	20.29% ± 0.28	23.19% ± 0.29	28.99% ± 0.31	126.20
Methanol crude	47.44% ± 0.35	53.85% ± 0.35	64.1% ± 0.33	82.05% ± 0.27	13.09
Aqueous crude	10.13% ± 0.21	11.39% ± 0.22	13.92% ± 0.24	26.58% ± 0.31	182.10

## Data Availability

Data is contained within the article.
